# Immune response to pneumococcal polysaccharides 4 and 14 in elderly and young adults. I Antibody concentrations, avidity and functional activity

**DOI:** 10.1186/1742-4933-2-10

**Published:** 2005-06-27

**Authors:** Kris Kolibab, S Louise Smithson, Anne K Shriner, Sadik Khuder, Sandra Romero-Steiner, George M Carlone, MA Julie Westerink

**Affiliations:** 1 Department of Medicine, Medical College of Ohio, Toledo, OH, USA; 2 Respiratory Diseases Branch, Division of Bacterial and Mycotic Diseases, National Centers for Infectious Diseases, Centers for Disease Control and Prevention, Atlanta, GA 30333, USA

## Abstract

*Streptococcus pneumoniae *is a serious worldwide pathogen and the focus of numerous vaccine development projects. Currently the most widely accepted surrogate marker for evaluating the efficacy of a given vaccine is to utilize ELISA. Measurement of antibody concentration by ELISA without reduction in cross-reactive antibodies causes an overestimation of antibody concentration and therefore protection, this is most notable in the aged, an at risk group for this infection. We compared the immune response to the pneumococcal polysaccharides (PPS) 4 and 14 of 20 young to 20 elderly adults. Pre-and post-vaccination IgG antibody concentrations and antibody avidity against PPS4 and PPS14 were measured using two different enzyme-linked immunosorbant assay (ELISA) absorption protocols. All sera were pre-absorbed with either cell-wall polysaccharide (CPS), or CPS and serotype 22F polysaccharide.

Pre- and post-vaccination IgG antibody concentrations for serotype 4, but not 14, were significantly lowered with the additional absorption with serotype 22F polysaccharide in both age groups. Young and elderly demonstrated a significant increase from pre- to post-immunization antibody concentration, using either absorption method; and opsonophagocytic antibody titers in response to both PPS4 and PPS14. The correlation coefficients between ELISA and opsonophagocytic assays were improved by additional absorption with serotype 22F in response to serotype 4, but not serotype 14 in all age groups. Opsonophagocytic antibody titers in a sub-group of elderly (>77 years of age) were significantly lower than the opsonophagocytic antibody concentrations in young adults.

These results suggest the importance of eliminating cross-reactive antibodies from ELISA measurements by absorption of serum and an age-related impairment in the antibody response to pneumococcal polysaccharides.

## Background

*Streptococcus pneumoniae *is a major human pathogen responsible for serious infections in all age groups worldwide. Pneumococcal infections are estimated to cause around 40,000 deaths per year [1], and are a leading causes of death in the United States [2]. The highest incidence of pneumococcal infections occurs at the extremes of age, in the very young and in the elderly. The mortality rate of invasive pneumoccoal disease increases with age and is estimated to be 20% in those 65 years of age and 40% in those 85 years of age or older. Moreover, *S. pneumoniae*, is the most common cause of community acquired pneumonia among the elderly [3] and the fifth leading cause of death in those 65 years and older [4,5].

Pneumococcal capsular polysaccharide (PPS) is a major virulence factor of *S. pneumoniae *and reduces phagocytosis in the host [6,7]. The currently licensed pneumococcal vaccine consists of 23 purified PPS serotypes, which account for 76–90% of IPD causing strains [8,9]. The capsular PPS vaccine is based on the observation that antibodies against the capsule protect against disease by enhancing complement dependent phagocytosis [6]. Pneumococcal vaccination is recommended for all persons 65 years and older in the United States, however, efficacy studies indicate decreased protection among this target population despite protective levels of anti-pneumococcal antibodies [10,11]. The underlying causes of the reduced vaccine efficacy merit investigation.

Reports of the diminished vaccine efficacy in the elderly may reflect either poor functionality of vaccine induced anti-PPS specific antibodies or inconsistent antibody measurements. Protection is measured by *in vitro *analysis of opsonophagocytosis by anti-polysaccharide antibodies and serves as a surrogate marker of clinical protection [12]. However, the opsonophagocytic assay is labor intensive and difficult to perform and therefore impractical, when analyzing large numbers of samples. Therefore, alternate methods for measuring pneumococcal antibody concentrations, namely ELISA, have been developed. More practical than opsonophagocytic assays, the polysaccharide-specific antibody concentrations measured by ELISA do not necessarily correlate with functional antibody activity. The development of an ELISA method that accurately reflects functional antibody activity and can serve as a surrogate marker of protection is of utmost importance.

The enzyme-linked immunosorbant assay (ELISA) method used to measure PPS-specific antibody concentrations has changed considerably over the past 20 years. The presence of cell wall polysaccharide (CPS), covalently linked to the type-specific polysaccharide, led to poor correlations between antibody concentration and pneumococcal vaccine efficacy [13,14]. The realization that the measurement of anti-CPS antibodies caused an overestimation of anti-PPS antibody concentration led to a modification of the pneumococcal ELISA procedure to include an absorption step, removing cell wall polysaccharide directed antibodies [13,15]. Recent studies have demonstrated that the additional absorption of sera with CPS and serotype 22F PPS significantly increases the correlation between antibody concentration and opsonophagocytic activity compared to CPS absorption alone [16].

Previously, a study performed in elderly adults demonstrated a significant decrease in opsonophagocytic activity in response to PPS4 despite normal antibody concentrations [17]. The decrease in functional antibody activity increased with age and was serotype specific. The immune response to PPS14, as determined by antibody concentration and opsonophagocytic antibody activity, was well conserved in the elderly. However, in this study, an older version of the PPS-ELISA relying solely on CPS absorption was used to determine antibody concentration. Therefore, non-specific cross-reactive antibodies may have been responsible for the discrepancy between antibody concentration and opsonophagocytic activity to PPS4.

The aim of this study was to re-assess the response of elderly (>65 years old) adults to PPS4 and PPS14 employing the recently modified PPS-specific ELISA. We have undertaken the present study to further elucidate the nature of the elderly immune response to PPS in an effort to define correlates of protection in the ageing population. Should the elderly response contain a significant proportion of low avidity, cross-reactive antibodies as previously suggested [17] one would expect that absorption with PPS 22F would have a greater impact on antibody concentration and avidity measurements in the elderly than young.

## Results

All volunteers were considered immune competent based on health history, complete blood cell counts, total immunoglobulin levels and quantitative B-cell and T-cell subset studies. There was no quantitative difference in number of B-cells or CD4+ and CD8+ T cells in the young versus elderly participants (Table 1).

**Table 1:** Measurement of T and B cell parameters in young and elderly volunteers.

	**CD4+ T cells GMN (95% CI)**	**CD8+ T cells GMN (95% CI)**	**B cells GMN (95% CI)**	**Total IgG GMC (95% CI)**
Normal Range	430 – 1185	180 – 865	25 – 285	591–1540
Young	879 (758–1000)	573 (487–659)	235 (156–314)	838 (757–919)
Elderly	817 (692–942)	396 (303–489)	164 (93–235)	993 (956–1030)

GMC = geometric mean concentration (ug/ml), GMN geometric mean number per ml. There was no significant difference (P>0.05) between young and elderly for all values.

### Anti-pneumococcal Antibody Concentrations

Anti-PPS4 and PPS14 antibody concentrations were determined by the two ELISA methods, absorption with either CPS or CPS + PPS22F. Geometric mean IgG antibody concentrations (μg/ml) and 95% CI are shown in Table 2.

**Table 2:** Specific anti-PPS IgG antibody levels in young and elderly.

**Pre-immune serum GMC (95% CI)**				
		**CPS**	**CPS + 22F**	**P value**
**PPS4**	Young	1.5 (0.59–2.41)	1.22 (0.77–1.66)	**0.033**
	Elderly	1.94 (0.85–3.03)	1.39 (0.97–1.81)	**0.008**
**PPS14**	Young	3.86 (2.54–5.18)	4.02 (2.10–5.94)	0.370
	Elderly	4.77 (1.61–7.93)	4.73 (2.42–7.04)	0.367

**Post-immune serum GMC (95% CI)**				
		**CPS**	**CPS + 22F**	**P value**

**PPS4**	Young	4.03 (1.68–6.38)	3.45 (1.62–5.28)	**0.036**
	Elderly	3.95 (0.73–7.17)	3.50 (1.01–5.99)	0.142
**PPS14**	Young	18.41 (7.24–29.58)	18.43 (8.46–28.40)	0.708
	Elderly	11.43 (4.44–18.42)	10.22 (3.35–17.09)	**0.013**

Geometric mean concentration (GMC, μg/ml) of specific anti-PPS IgG antibody in pre-and post-immunization serum from young and elderly volunteers by two ELISA methods. CPS – serum adsorbed with CPS prior to ELISA. CPS+22F – serum absorbed with PPS22F and CPS prior to ELISA. P values shown in bold indicate significant difference between absorption methods by two tailed paired Student's T-test.

In response to PPS4 a significant increase in pre-to post-immunization antibody concentration was detected by both ELISA methods in both age groups (p range 0.003 – 0.005). When both ELISA methods were compared a significant reduction in antibody concentration was observed following CPS + PPS22F absorption in young volunteers' pre-and post-immune sera (18.6 %, p = 0.033 and 14%, p = 0.036 respectively) and elderly volunteers' pre-immune (28.4, p = 0.008) but not post-immune sera (11.4%, p = 0.142). There was no significant difference in the antibody levels detected between young and elderly volunteers in pre-or post-immunization sera using either absorption method, although young pre-immune sera tended to demonstrate lower geometric mean concentrations (GMC). However, more young than elderly demonstrated a two-fold or greater increase pre-post immunization (83% compared to 50%).

In response to PPS14, a similar significant increase in pre-to post-immunization antibody concentration was detected in all groups (p range 0.001–0.006). When both ELISA methods were compared there was no significant difference in IgG concentrations in young sera using either method. However, in the elderly the addition of PPS22F absorption significantly reduced the IgG concentration detected in post-(10.6%, p = 0.013) but not pre-immunization sera (0.8%, p = 0.367). There was a tendency towards lower antibody concentrations in the pre-immune sera of the young in comparison to the elderly, although this was not significant. The post-immune anti-PPS14 antibody concentrations were also not significantly different between young and elderly groups. However, more young than elderly demonstrated a two fold or greater increase in serum IgG concentrations, 90% and 55% respectively.

Our study was designed to compare the immune reaction to PPS in young adults (18 – 30 years old) to elderly (>65 years of age). However, several studies [17,18] indicate that the oldest elderly (>77 years of age) demonstrate a significant decrease in the immune response to PPS. We thus analyzed the results obtained for a subgroup of elderly >77 years old within our study population. In this small subgroup (n = 6) additional absorption with PPS22F resulted in a substantial but not significant decrease in measured antibody concentration of 28% in pre-immune and 15.6% in post-immune sera in comparison to young adults. A significant increase from pre-to post-immunization antibody concentration in response to PPS4 was only found when sera were absorbed with CPS (p = 0.05) but not when sera were absorbed with CPS + PPS22F (p = 0.069). There was no significant increase in pre-to post-immunization antibody levels against PPS14 for either method (p = 0.094 for both). Although, the post-immunization anti-PPS14 IgG GMC of the young adult group was not significantly different from the elderly, it was significantly higher than the sub-group of elderly >77 years old using both absorption methods (p = 0.035 and p = 0.022). There was a reduction in the proportion of this subgroup that demonstrated a two-fold or greater increase in IgG levels pre-post immunization, in response to both PPS4 (29%) and PPS14 (43%) than either all elderly (50% and 55% respectively) and young (83% and 90% respectively).

### Immunoglobulin Isotype and Subclass

The predominant immunoglobulin isotype expressed in response to both PPS4 and PPS14 consisted of IgG. Despite a significant increase from pre-to post-immunization, the GMC of the PPS-specific IgM and IgA antibody concentrations in response to both PPS did not exceed one microgram per millilitre in young or elderly. Moreover, there was no significant difference in either IgM or IgA antibody concentrations in young versus elderly. Additional absorption with PPS22F reduced both IgM and IgA concentrations in young and elderly alike, but not significantly so.

As shown in Table 3, the IgG response to PPS4 and PPS14 was dominated by the IgG2 subclass in young and elderly adults as previously reported [19,20]. In response to PPS4, 67.5 to 80.5 % of the total IgG antibody concentration measured consisted of IgG2, with no significant difference between young and elderly or pre-to post-immunization. Similarly, of the pre-and post-immune total PPS14-specific IgG antibody, 63.7 to 73% of the response consisted of IgG2 with no significant difference between age groups.

**Table 3:** Anti-PPS specific IgG isotype levels in young and elderly.

		**Pre-immune, GMC (95% CI)**	**Post-immune GMC (95% CI)**	**P value**
**IgG1**				
**PPS4**	Young	0.13 (0.06)	0.35 (0.18)	**0.001**
	Elderly	0.14 (0.09)	0.33 (0.38)	0.051
**PPS14**	Young	0.87 (0.13)	3.59 (0.33)	**0.001**
	Elderly	0.93 (0.44)	1.19 (0.95)	**0.015**

**IgG2**				
**PPS4**	Young	1.03 (0.37–1.69)	5.14 (0.64–9.64)	**0.010**
	Elderly	0.83 (0.04–1.62)	1.96 (0.98–2.94)	**0.005**
**PPS14**	Young	2.18 (1.86–2.50)	12.33 (4.51–20.15)	**0.002**
	Elderly	1.36 (0.62–2.10)	4.78 (2.37–7.19)	**0.004**

Geometric mean concentration (GMC μg/ml) of anti-PPS antibody in pre-and post-immunization serum from young and elderly volunteers measured following absorption with CPS. P values shown in bold indicate significant difference between pre and post immunization by two tailed paired students T-test

PPS-specific IgG1 antibodies were also readily detectable and increased significantly post-vaccination in both age groups. Overall, IgG1 antibodies represented 9.5 to 11.2% of total IgG antibodies in response to PPS4 and 18.7 to 25.4% of total IgG antibodies measured in response to PPS14. There was no significant difference detected in either IgG1 antibody concentration or percent usage between young and elderly.

Additional absorption of sera with PPS22F did not affect either pre-or post-immunization IgG1 PPS4 or PPS14 concentrations in either age group or the IgG2 response to PPS14. However, in response to PPS4, absorption with PPS22F significantly reduced pre-and post-immunization IgG2 concentrations in both young and elderly (17 to 27% with p = 0.019 to 0.025).

### Opsonophagocytic Activity

Opsonophagocytic activity was measured in pre-and post-immunization sera against pneumococcal serotypes 4 and 14. Geometric mean titers (GMT) and ranges are shown in Table 4.

**Table 4:** Opsonophagocytic activity in young and elderly.

		**Pre-immune GMT (95% CI)**	**Post-immune GMT (95% CI)**	**P value**
**PPS4**	Young	10.2 (4.43–15.97)	93.6 (-19.63–206.89)	**0.001**
	Elderly	14.9 (2.74–27.12)	147.0 (-16.63–310.70)	**0.001**
**PPS14**	Young	16.7 (-33.03–66.36)	245.8 (82.50–409.05)	**0.0004**
	Elderly	26.9 (-104.78–158.60)	265.03 (115.74–414.32)	**0.0003**

Geometric mean titer of serotype specific opsonophagocytic activity in pre-and post-immunization serum, from young and elderly volunteers. Significance of increase from pre to post-immunization was calculated using two tailed paired students T-test.

In response to serotypes 4 and 14 both young and elderly demonstrated a significant increase pre-to post-immunization (p range 0.0003–0.001). There was no significant difference between the opsonophagocytic titers in young and elderly for either polysaccharide (p range 0.203–0.754). However, when the eldest subgroup was analyzed separately there was a significant increase in opsonophagocytic activity only in response to serotype 14 (p = 0.04) but not in response to serotype 4 (p = 0.214). The post immunization titers for the eldest subgroup for serotype 4 (GMT 35.92) and 14 (90.51) were significantly lower than in young donors (p = 0.03 and 0.02), all elderly donors (p = 0.01 for both) and the younger group of elderly (p = 0.002, 0.001).

### Antibody Avidity Measurements

Antibody avidity was determined using sodium thiocyanate (NaSCN) in both ELISA methods as described. Geometric mean concentrations (GMC, M NaSCN) and ranges are given in Table 5. The addition of PPS22F to the assay did not significantly change the avidity measurements, pre-or post-immunization, in young or elderly (p range 0.053 – 0.715) except in young anti-PPS14 post immunization sera (p = 0.04). In response to PPS4 there was no significant difference between young and elderly in any group analyzed (p range 0.32 – 0.93). In response to PPS14 a significant difference was detected between young and elderly pre-immune sera when absorbed with CPS alone (p = 0.03) and post-immune sera when absorbed with CPS and PPS22F (p = 0.03).

**Table 5:** Avidity of anti-PPS antibodies from young and elderly.

**Pre-immune serum GMC (95% CI)**				
		**CPS**	**CPS + 22F**	**P value**
**PPS4**	Young	0.19 (0.07–0.32)	0.21 (0.04–0.38)	0.091
	Elderly	0.22 (0.13–0.31)	0.22 (0.12–0.31)	0.646
**PPS14**	Young	0.28 (0.21–0.36)	0.27 (0.19–0.35)	0.711
	Elderly	0.43 (0.27–0.59)	0.42 (0.27–0.56)	0.196

**Post-immune serum GMC (95% CI)**				
		**CPS**	**CPS+22F**	**P value**

**PPS4**	Young	0.35 (0.22–0.49)	0.40 (0.25–0.55)	0.053
	Elderly	0.43 (0.23–0.63)	0.46 (0.26–0.65)	**0.014**
**PPS14**	Young	0.47 (0.33–0.62)	0.41 (0.29–0.54)	0.618
	Elderly	0.68 (0.50–0.85)	0.66 (0.48–0.84)	0.715

Geometric Mean Concentration (GMC, M NaSCN) representing serum avidity by two different ELISA methods. CPS – serum adsorbed with CPS prior to ELISA. CPS+22F – serum absorbed with PPS22F and CPS prior to ELISA. P values shown in bold indicate a significant difference between absorption methods calculated using paired Student's T-test.

Anti-PPS4 antibodies demonstrated a significant increase in avidity pre-to post-vaccination in elderly volunteers by both methods (p = 0.01, 0.003) but not in the young (p = 0.147, 0.257). Young anti-PPS14 antibodies showed a significant increase pre-to post-immunization when sera were absorbed with CPS alone (p = 0.015) but not CPS and PPS22F (p = 0.061). Avidity of elderly anti-PPS14 antibodies pre-and post-immunization was significantly different by both methods (p = 0.007, 0.002).

In the oldest subgroup of elderly anti-PPS4, but not anti-PPS14, antibodies, when absorbed with CPS and 22F demonstrated a significant increase pre-to post-immunization (p = 0.03). There was no significant difference between avidity measurements in this subgroup compared to either young, all elderly or the youngest elderly.

### Correlation between Opsonophagocytic Antibody Activity and Antibody Concentrations

Correlation coefficients of association (*r *values) were determined between log_2 _IgG antibody levels and log_2 _opsonophagocytic titers for PPS4 and PPS14 by absorbing sera with CPS or CPS and PPS22F (Table 6). In response to PPS4, the correlation coefficient increased following CPS+ PPS22F absorption in young adults from an *r *value of 0.64 (CPS absorption) to 0.81 (CPS+ PPS22F absorption). A similar increase from 0.69 with CPS and 0.79 with CPS+PPS22F absorption was noted in the elderly. The sub-group of elderly >77 years of age showed a correlation coefficient of 0.73 following absorption with CPS that increased to 0.87 following absorption with CPS and PPS22F. Thus, all age groups demonstrated a significant correlation between antibody concentration and opsonophagocytic antibody response that improved by additional absorption with CPS and PPS22F.

**Table 6:** Correlation between opsonophagocytic titers and antibody concentration.

		**CPS**	**CPS + PPS22F**	**P value**
**PPS4**	Young	0.64	0.81	0.28
	Elderly	0.69	0.79	0.51
**PPS14**	Young	0.90	0.88	0.78
	Elderly	0.75	0.72	0.85

Correlation coefficients of association (*r *values) between post-immunization opsonophagocytic titer and antibody concentration following absorption with CPS or CPS + 22F in young and elderly volunteers. There was no significant difference between absorption techniques by Fisher's Z transformation.

In addition, all age groups demonstrated a relatively high correlation between antibody concentration and functional antibody activity in response to PPS14. Additional absorption with PPS22F did not improve the correlation coefficients of association between IgG antibody concentrations and opsonophagocytic titers for PPS14. All age groups showed a significant correlation between antibody concentrations and opsonophagocytic antibody activity as previously reported in adults [16].

### Correlation between Opsonophagocytic Antibody Activity and Antibody Avidity

We also determined the correlation coefficients of association (*r *values) between concentration of NaSCN necessary to reduce ELISA optical density by 50% and log_2 _opsonophagocytic titers for PPS4 and PPS14. In response to PPS4, all groups showed a relatively poor correlation between avidity and opsonophagocytic activity (Table 7) that improved marginally after absorption with CPS and PPS22F. In the sub-group of elderly >77 years the correlation of association was very poor, however the *r *value improved markedly from 0.069 to 0.25 by additional absorption with PPS22F.

**Table 7:** Correlation between opsonophagocytic titers and antibody avidity.

		**CPS**	**CPS + PPS22F**	**P value**
**PPS4**	Young	0.39	0.44	0.86
	Elderly	0.52	0.53	0.97
**PPS14**	Young	0.51	0.49	0.94
	Elderly	0.64	0.59	0.81

Correlation coefficients of association (*r *value) between post-immunization opsonophagocytic antibody titer and antibody avidity following absorption with CPS or CPS + 22F in young and elderly volunteers. There was no significant difference between absorption techniques by Fisher's Z transformation.

In response to PPS14, the correlation coefficients of association were better than those in response to PPS4 (Table 7). The additional absorption with PPS22F however, did not significantly affect the correlation coefficients of association.

## Discussion

Recent improvements in the ELISA method used to measure anti-pneumococcal polysaccharide-specific antibodies, aimed at removing non-specific antibodies, have resulted in a significant increase in the correlation between antibody concentration and opsonophagocytic activity [16]. Although this methodology has been extensively evaluated in healthy adults [16] the effect of additional serum absorption with PPS22F on the measured antibody response in elderly adults has not been determined. The presence of non-serotype specific, poorly functional antibodies may in fact explain the discrepancy between measured antibody concentrations and functional antibody activity in the elderly.

The results of the present study indicate that both young and elderly adults respond to pneumococcal vaccination with a significant increase in PPS-specific antibody concentration, although the fold increase in antibody concentration was lower in elderly. Additional absorption with PPS22F resulted in a significant reduction in antibody concentration to PPS4 but did not affect the antibody concentration measurements to PPS14. It is likely that PPS14 does not contain cross-reactive antigens or contaminants as described for other pneumococcal polysaccharides [16,21,22]. The decrease in measured anti-PPS4 antibody resulted in an increased correlation between antibody concentration and functional antibody activity, consistent with previous studies [16]. Although the decrease in anti-PPS4 antibody was more pronounced in pre-immune sera, it was not significantly different between young and all elderly and did not affect fold-increase in antibody concentration post-immunization. The results of previous studies [18,23] however, indicate that an impaired immune response to PPS is limited to the oldest elderly. Although small in number (n = 6), separate analysis of elderly > 77 years of age showed a decrease in significance of the increase from pre-post when the sera was absorbed with PPS22F. In addition fewer volunteers in this age group demonstrated a two fold or greater increase in antibody concentration pre-post than either the young or elderly. These results suggest that previous studies may have shown an overestimation of antibody concentration and that this may be particularly critical in the oldest elderly population. The additional absorption with PPS22F thus revealed a potential impairment in quantitative antibody response to PPS4 in the oldest age group.

Several studies have demonstrated that reduced or absent functional antibody activity is directly related to low antibody avidity and not necessarily a result of low antibody concentration [24,25]. Specifically, it has been demonstrated that post-vaccination sera from elderly with low opsonophagocytic activity correlate with low IgG antibody avidity [23]. Additional absorption with PPS22F, which serves to remove non-specific antibodies with presumably low avidity, should in principle result in increased avidity measurement. In response to PPS4, absorption of sera with CPS and PPS22F increased antibody avidity in young and elderly adults, although this increase was only significant in the young. However, the moderate correlation between opsonophagocytic antibody titers and antibody avidity in both young and elderly, did not improve significantly following absorption with PPS22F. These data suggest that high avidity antibodies are not necessarily functional, or conversely, opsonophagocytic antibodies are not necessarily of high avidity. In contrast, in response to PPS14, there was a strong correlation between opsonophagocytic antibody titers and antibody avidity in all age groups, including elderly > 77. Similarly, Romero-Steiner, et al. [23] found a significant correlation between serum opsonophagocytic activity and antibody avidity, particularly in response to PPS14. In most elderly individuals, specifically those > 77 years of age, low opsonophagocytic antibody activity was directly related to low antibody avidity.

Opsonophagocytic activity is dependent on both antibody avidity and antibody concentrations. In the elderly, particularly those > 77 years old, lack of correlation between functional antibody activity and antibody avidity was generally attributable to low antibody concentrations. In contrast, some of the young volunteers with low opsonophagocytic activity demonstrated moderate to high antibody concentrations with moderate or high antibody avidity. These data suggest that young adults in particular, may generate high levels of high avidity antibodies, not directed at opsonophagocytic epitopes. Based on these data, we propose that at least two different mechanisms may be responsible for the discrepancy between antibody concentration and functional antibody activity, namely, poor antibody avidity and antibodies directed at non-opsonophagocytic epitopes present on the pneumococcal polysaccharide.

While overall there was no statistical difference between the response in young and elderly there was a trend towards a reduced response which increased with age. Less of the eldest subgroup of elderly responded to the vaccine with a two fold or greater concentration of antibody than either all elderly or young. Immunosenescence is an ongoing process with age; as the patient ages the immune system continues to decline. Thus, in studies aimed at detecting the most differences with ageing, future studies should aim to enroll older elderly, i.e. those over 75 years of age.

## Conclusion

Our study indicates that additional absorption of serum with PPS22F results in an improved correlation between measured antibody concentration and functional antibody activity in young and elderly adults. The removal of non-specific antibodies may, however, be of particular importance in assessing PPS-specific antibody concentration in elderly and may uncover previously un-noted differences in quantitative antibody response.

## Methods

### Human Volunteers/Vaccination

Healthy young adults (<30 years old) and elderly volunteers (>65 years old) were asked to participate in this study. Elderly volunteers were recruited from the community as well as from the general internal medicine clinic at Medical College of Ohio. Young volunteers were recruited from the student population at MCO. Each individual was questioned concerning prior pneumococcal vaccination, medications, previous illness and present health. In addition, we obtained complete blood count (CBC), comprehensive chemistry profile including renal and liver functions and serum albumin, total B cells, T cell subsets and total IgG, IgM and IgA levels in all study candidates. Individuals previously immunized with the pneumococcal vaccine and any individual considered to be immunocompromised on basis of medication (chemotherapy, steroid preparations, immunosuppressive agents including anti-TNFa agents), previous/present illness (previous pneumococcal disease, splenectomy, auto-immune disease, end-stage renal or liver disease, HIV positivity, organ transplant or cancer) and those with abnormal CBC, chemistries, B or T cells or immunoglobulin levels did not qualify. Informed consent was obtained from all participants using protocols reviewed and approved by Institutional Review Board of Medical College of Ohio.

### Pneumococcal Polysaccharide ELISA

ELISA was used to determine the anti-PPS specific human antibodies, isotypes and subclasses, in all volunteers before and 6 weeks after vaccination [23,26]. Comprehensive details regarding this procedure are found in the web document: . US reference Pneumococcal Antiserum 89SF, courtesy of Dr Carl Frasch CBER/FDA, was used as a control throughout.

Briefly, Nunc microtiter plates were coated with PPS4 or PPS14 (ATCC, Rockville, Md.). Human sera were absorbed with 5 μg/ml of cell wall polysaccharide (CPS) or with 5 μg/ml of CPS and 10 μg/ml of serotype 22F polysaccharide (ATCC) and incubated for 30 minutes [26]. Serial dilutions of absorbed sera were added to the wells and incubated. Horse-radish peroxidase labeled anti-human isotype and subclass specific conjugates were added and reaction developed with σ-phenylenediamine (Sigma, St. Louis), absorbance was read at 490 nm.

### Antibody Avidity ELISA

Avidity of IgG antibodies to PPS4 and PPS14 was determined by sodium thiocyanate (NaSCN), a chaotropic agent that interferes with antigen-antibody interaction [27]. The molarity of NaSCN required to elute 50% of bound antibody was used as a measure of avidity, the relative strength of antigen-antibody binding. ELISA was performed as previously described [23]. Microtiter plates were set up with NaSCN molarity ranges from 4 M to 0.0625 M. Controls consisted of uninhibited serum samples and blanks. All serum samples were tested in duplicate. Avidity was expressed as the molarity of NaSCN required to inhibit 50% of binding of uninhibited serum.

### Opsonophagocytic Assays

Opsonophagocytic assays were performed as previously described [23,28] to measure the functional antibodies against PPS4 and PPS14. Briefly, viable pneumococci were allowed to pre-opsonize with serially diluted heat-inactivated sera. Newborn rabbit serum (Pel-Freez, Brown Deer, WI) was added as source of complement (12.5%). Washed, differentiated cells were added at a 400:1 effector: target cell ratio. After incubation, to allow phagocytosis to occur, an aliquot was plated and incubated overnight at 37°C with 5% CO_2_. The opsonophagocytic titer was determined as the reciprocal of the dilution with >50% killing when compared to the control wells without serum.

### Statistics

Geometric mean concentrations of IgG reactive with PPS4 and PPS14 were calculated for each group. Pre-to post-immunization values were compared using paired Student's T test, young and elderly were compared using two sample, unpaired Student's T test. Correlation coefficients and p values were calculated using Fisher's Z transformation using log_2 _base of opsonophagocytic titers and antibody concentrations. P values less than 0.05 were considered to be significant. Statistical calculations were performed with use of SPSS software 11.5.1.

## Competing interests

None of the authors of this paper have a commercial or other association that might cause conflict of interest.

## Authors' contributions

KK: performed ELISAs, collated data, wrote initial draft of manuscript

SLS: adapted assays for laboratory, critical revision of manuscript, final revision of manuscript

AKS: performed opsonophagocytic assays

SK: provided key statistical help and advice

SRS: consultant regarding opsonophagocytic assay, provided cell lines and controls

GMC: consultant regarding ELISA assay, provided critical controls

MAJW: conceived of the study, critical revision of manuscript
